# Assessing the burden of dengue among household members in Alaminos, Laguna, the Philippines: a prospective cohort study

**DOI:** 10.2478/abm-2021-0027

**Published:** 2021-10-29

**Authors:** Maria Rosario Capeding, Melanie de Boer, Silvia Damaso, Adrienne Guignard

**Affiliations:** Department of Microbiology, Research Institute for Tropical Medicine, Muntinlupa, 1781 Metro Manila, Philippines; Vaccines, GSK, Rockville, MD 20850, USA; Vaccines, GSK, Wavre 1300, Belgium

**Keywords:** dengue, epidemiology, Philippines, sentinel surveillance, reverse transcriptase-polymerase chain reaction

## Abstract

**Background:**

The incidence of dengue is increasing rapidly and is a challenging health issue in the Philippines. Epidemiological data are largely based on a passive-surveillance reporting system, which leads to substantial under-reporting of cases.

**Objectives:**

To estimate dengue infection and disease incidence prospectively at the community level in an endemic area of the Philippines using an active surveillance strategy.

**Methods:**

We implemented active surveillance in the highly endemic community of Alaminos, Laguna. The study consisted of a 1-year follow-up with 2 visits scheduled at the start and end of the study, as well as regular active surveillance in between and unscheduled visits for suspected cases. Blood samples were collected and analyzed to detect dengue during the first scheduled visit and all unscheduled visits, and clinical examination was performed at all visits (registered at clinicaltrials.gov NCT02766088).

**Results:**

We enrolled 500 participants, aged from 6 months to 50 years; 76.2% were found positive for immunoglobulin G (95% confidence interval [CI], 71.9–80.0), with 92.0% among those aged 9–17 years. Active (weekly) surveillance identified 4 virologically confirmed cases of dengue (incidence proportion 0.8; 95% CI 0.3–2.1); all in participants aged ≤14 years.

**Conclusions:**

Routine surveillance programs such as sentinel sites are needed to characterize the entire clinical spectrum of symptomatic dengue, disease incidence, and transmission in the community.

Dengue is a mosquito-borne disease caused by the 4 serotypes of the single-stranded RNA dengue virus (DENV-1, DENV-2, DENV-3, and DENV-4) [1]. The clinical symptoms of the disease develop in 3 phases: first an acute febrile phase with sudden high-grade fever lasting for 2–7 days, occasionally followed by clinically substantial plasma leakage lasting for 1–2 days (critical phase) with increased capillary permeability and hematocrit levels [2]. Most patients recover after this phase, but some progress to severe disease with plasma leakage resulting in shock (dengue shock syndrome), with severe bleeding or organ failure [2]. If untreated, dengue may cause death in 20% of patients. This mortality drops to <1% if adequate clinical management is offered in severe cases [3].

Worldwide, the incidence of dengue is rapidly increasing, 15-fold in the past 20 years; half of the global population remains at risk, and the World Health Organization estimates 100–400 million worldwide infections every year [4]. Dengue is an important health issue in the Philippines and for all regions of the world with tropical and subtropical climates [5, 6]. A study assessing the dengue burden in the Philippines estimated nearly 800,000 episodes of dengue per year, based on 2010–2014 surveillance data [7]. Moreover, in Punta Princesa, Cebu City, the monthly dengue infection incidence rate per 1000 population ranged from 0.0 to 3.25 based on active surveillance of a prospective cohort analysis and from 0.18 to 0.44 based on Cebu City Health Department passive surveillance data [7]. Similarly, a dynamic-transmission model estimated that between 2016 and 2020, >400,000 patients with cases of dengue would be hospitalized and about 240,000 would be treated as outpatients; all for an average annual aggregated cost of USD 158 million for hospitalized and ambulatory patients, and USD 19 million in productivity losses [8]. While Asia bears most of the global disease burden, the disease endemicity is also present in the Western Pacific, the Americas, Africa, and the Eastern Mediterranean, and the infection continues spreading in previously unaffected regions of the world [4, 6]. Dengue outbreaks are seasonal, influenced by characteristics of the vector and the host [2, 9]. Moreover, the incidence of dengue shows cyclical variations from highly epidemic to nonepidemic years [2]. The year 2019 was so far the peak year for worldwide reported dengue cases, and the worst in the recorded history of the Philippines, with 420,000 dengue cases being reported that year [4].

The global increase in dengue infections is believed to be due to population growth, increasing worldwide mobility, urbanization, climate change, and the inability to sustain effective vector control [3, 10]. The increase is also explained partly by an acquired increased awareness by national governments, which has led to improvements in surveillance and reporting. However, because we are still lacking accurate burden estimates, available estimates merely suggest the actual incidence [4]. There is considerable under-reporting of mild dengue cases in parallel with a great number of misdiagnoses as “other” febrile illnesses [4, 7, 11,12,13]. Passive routine surveillance systems, currently the main systems used to monitor dengue, do not capture a large proportion of cases [7, 11, 13]. In the Philippines, a study comparing active with passive dengue surveillance found that active surveillance symptomatic dengue episodes were 4.7 times higher than the number of cases reported by passive surveillance [7]. Passive surveillance provides only a partial estimate of the burden of dengue because it does not detect inapparent infections, which represent most cases and contribute to disease transmission. Active surveillance is considered valuable as a method to understand the prevalence of the DENV serotypes, using serotype-specific polymerase chain reaction (PCR) for detection [14]. PCR can be complemented by immunological tests for the nonstructural protein 1 (NS1) or serological assays for the diagnosis of patients presenting >5 days after onset of symptoms, although these assays do not allow the serotype identification. Early detection of dengue would allow the prompt implementation of management strategies, which are particularly important in highly endemic areas [15]. Estimates of inapparent cases and virologically confirmed clinically apparent cases will improve the understanding of the disease burden and assist in formulating prevention strategies and conducting vaccine clinical trials [16].

Only one vaccine, a tetravalent live-attenuated chimeric yellow fever dengue vaccine, (*Dengvaxia*, Sanofi Pasteur), is currently available and is licensed in about 20 countries [4]. However, because this vaccine imposes a risk for severe dengue in seronegative individuals, its use is limited to sero-positive individuals with a history of dengue infection, who are also living in endemic areas and are aged 9–45 years [4, 17]. To make progress with further dengue vaccine development programs, it is important to achieve an understanding of disease dynamics at the community level. In light of this, the present observational cohort study was conducted to estimate infection and disease incidence of dengue at the community level in an endemic area, as is Alaminos, Laguna, in the Philippines [5]. Specifically, the primary objective of this study was to estimate the overall incidence of symptomatic dengue cases confirmed by reverse-transcriptase quantitative PCR (RT-qPCR). The secondary objectives were (a) to estimate by age, sex, serotype (if applicable) and DENV immunoglobulin G (IgG) serological status at enrollment, the incidence of virologically confirmed dengue cases (by RT-qPCR or by the NS1 assay), and the incidence of probable dengue cases, (b) to estimate the prevalence of anti-DENV (Ig) antibodies at enrollment, overall and by age, and (c) to describe clinical presentations of confirmed and probable dengue cases.

## Methods

### Study design and setting

This was a multicenter, prospective, household-based cohort surveillance study conducted in geographically defined communities in Latin America and Southeast Asia. Here we report on the data obtained between September 2017 and December 2018 in Alaminos, Laguna, the Philippines.

The study was conducted following Good Clinical Practice and all applicable regulatory requirements including the contemporary revision of the Declaration of Helsinki and was approved by the Institutional Review Board of the Research Institute for Tropical Medicine of the Department of Health at the Muntinlupa City, on February 16, 2016. The study was registered at www.clinicaltrials.gov (Clinical Trial Registration No. NCT02766088).

Study researchers used public announcements and community meetings to inform the community about the study. Eligible individuals were aged from 6 months to 50 years, living in households reachable by phone who planned to remain at the same residence throughout the study, and who agreed to go to the study site for visit(s) in case of acute febrile illness. They were also required to observe and document the signs and symptoms of dengue, and required to understand how to take and report body temperature. Informed consent to participate was obtained from all eligible individuals or their legal guardians and documented.

A maximum of 2 people per household was considered sufficient to capture the prevalence of infection among household members in the community. The recruitment was a collaboration between the study team and the Municipal Health Office. Basic information about the study (i.e., study title, the eligible age for participants, required consent, site of the study) was given by the health center staff to visiting parents of possible participants, and posters containing basic study information had been displayed at collaborating health centers. Parents were invited to visit the study site if interested in additional information.

### Study procedures

**Figure 1:**
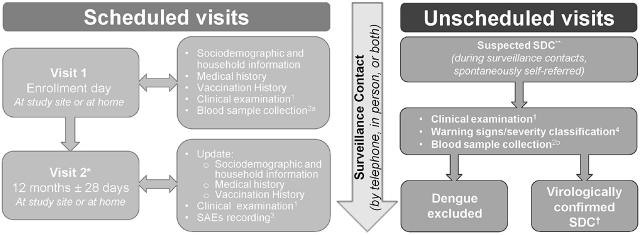
Schematic representation of the study design. *Blood samples were collected at Visit 2, but not analyzed due to early study termination. **Body temperature ≥ 38.0 °C within the past 8 days lasting from 36–48 h to 7 days, potentially accompanied by other signs of dengue that by the study investigator’s opinion could only be related to dengue; †SDCs confirmed by RT-qPCR or NS1. **1.** Detailed clinical examination assessing the participant’s general condition, cardiac and respiratory rates, blood pressure, dengue-associated clinical signs, and symptoms. **2a.** Used for ELISA to detect anti-DENV indirect IgG. **2b.** Used for **i.** DENV RT-qPCR, **ii.** DENV isolation for sequencing purposes, **iii.** DENV sequence, **iv**. DENV NS1 rapid test (ICT) or ELISA, **v.** IgM/IgG rapid test (ICT) or *SD Bioline Dengue Duo* (dengue NS1 antigen and IgG/IgM). **3.** Participants were advised to contact study investigators to report any sign or symptom they perceived as an SAE. All SAEs related to study procedures (blood collection) were recorded and evaluated by the study investigator along with related signs, symptoms, and relevant clinical information. **4. A. Warning signs**. At least one of the following should be present: abdominal pain or tenderness, persistent vomiting, clinical fluid accumulation, mucosal bleeding, liver enlargement, increase in hematocrit concurrent with a rapid decrease in platelet count, lethargy, restlessness. **B. Criteria for severe dengue**. Dengue with at least one of the following: severe plasma leakage leading to shock, fluid accumulation with respiratory distress, severe bleeding, severe organ involvement, failure of heart and other organs. DENV, dengue virus; ELISA, enzyme-linked immunosorbent assay; ICT, immunochromatographic assay; IgG, immunoglobulin G; IgM, immunoglobulin M; NS1, nonstructural protein 1; RT-qPCR, reverse-transcriptase quantitative polymerase chain reaction; SAE, serious adverse event; SDC, suspected dengue case.

The follow-up was originally planned to last 2 years. However, the study was prematurely terminated by the sponsor at 12 months of follow-up because in December 2017 the sponsor had decided to deprioritize the development of the dengue purified inactivated vaccine candidate to which the study was related. Therefore, only 2 scheduled visits were made (**Figure 1**): the first at enrollment (Visit 1) and the second at 12 months (Visit 2). Between these visits, surveillance for febrile illness was performed regularly, preferably once a week by telephone or in person, including a home visit at least every other week. In the event of fever, information was collected by telephone; 3 call attempts were made by the study nurse at 2 different times during the first day and another call was made on the next day. Unscheduled visits to the study site were required as soon as possible in case of suspected dengue case (SDC); preferably within 5 days following the onset of symptoms (**Figure 1**). Visit procedures are outlined in **Figure 1**.

Blood samples for laboratory assays were collected at Visit 1 and at all unscheduled visits (**Figure 1**). Blood samples were collected in serum-separation tubes (BD SST Gold tubes, catalog No. 367983 (3.5 mL) or 367986 (5.0 mL)). At scheduled visits for patients aged from 2 months to 2 years, 3.5 mL of blood was collected and for those aged from 2 years to 50 years, 2 × 5 mL of blood was collected. Blood was centrifuged at room temperature to obtain serum, which was allocated as follows: 0.2 mL (1.8 mL Nunc cryovial) for a dengue IgG enzyme-linked immunosorbent assay (Dengue IgG Indirect ELISA; Panbio) [18] and 1 mL (1.8 mL Nunc cryovial) for a DENV neutralization assay, and the remainder for tertiary assays (not conducted). All serum samples were frozen at −20 °C before being sent on dry ice to another laboratory for testing. Assays for the diagnosis and DENV types were determined using standardized and qualified or validated procedures.

At SDC visits, for early presenters, blood was similarly collected and serum similarly prepared and allocated for the assays as follows: 0.75 mL (1.8 mL Nunc cryovial) stored at −70 °C and sent on dry ice to another laboratory for RT-qPCR (*Simplexa Dengue Kit*, Focus Diagnostics) [19]. This assay amplifies 4 serotype-specific regions (Dengue 1: NS5 gene; Dengue 2: NS3 gene; Dengue 3: NS5 gene; Dengue 4: capsid gene) that allow serotype discrimination [19]. A second aliquot of 0.5 mL (1.8 mL Nunc cryovial) was used for a rapid (point-of-care) immunochromatographic diagnostic test for dengue NS1 and immunoglobulin M (IgM) (*SD Bioline Dengue Duo*, Abbott) [20] used to detect simultaneously both the NS1 antigen and the differential IgG/IgM DENV antibodies. If not available 2 tests were performed to obtain a combined result using the enzyme-linked immunosorbent assay (ELISA) (Panbio) [18] and a one-step sandwich format microplate enzyme immunoassay (*Platelia Dengue NS1 Ag*, Bio-Rad) for the qualitative or semiquantitative detection of DENV NS1 [21]. The rapid tests provided prompt laboratory results to the study investigator and study participants. Likewise, they also provided information on the diagnosis in late presenters, that is, >6 days from the onset of symptoms, for whom RT-qPCR has a lower probability of detecting the virus. For late presenters, blood was similarly collected and serum similarly separated, but only 0.5 mL of serum was aliquoted for onsite testing for specific IgM/IgG and NS1 as described above, RT-qPCR was not usually conducted. The remainder of serum was frozen in 1.8 mL Nunc cryovials at −20 °C for tertiary assays (not conducted).

### Definition of cases

A study participant was considered to have a SDC in the presence of body temperature ≥38.0 °C lasting from 36–48 h to 7 days within the past 8 days, accompanied by other signs and symptoms that the study investigator felt could be related to dengue. These other dengue symptoms or signs might not have had a defined focus or an obvious reason unrelated to dengue (based on the judgment of the physician) and include upper respiratory tract and/or gastrointestinal symptoms, headache, joint ache, and others. A *virologically confirmed dengue case* was a SDC confirmed by either RT-qPCR or NS1. A *probable SDC* was negative or RT-qPCR or NS1 assay not performed and was (a) DENV antibody IgM positive, or (b) was DENV antibody IgG positive.

### Statistical methods

#### Sample size calculation

The sample size calculation was performed at the multicenter level of the study, and with a target population of 500 for the Philippine site. The target population was estimated at 1750 overall participants (approximately 300 to 500 per site). A dropout rate of 5% per year was estimated, which would lead to approximately 1662 participants completing the first year of follow-up and approximately 1579 completing 2 years of follow-up, accumulating 3327 patient years.

The incidence of dengue was likely to vary by site and by age group. The study population should thus include between 30% and 50% of adults, but the distribution was not further specified as it would make operational feasibility more complex.

Supposing that the study population was composed of 70% of children with an expected incidence of 8 RT-qPCR-confirmed dengue cases per 1000 person–years and 30% of adults with an expected incidence of 5 RT-qPCR-confirmed dengue cases per 1000 person–years, the study would detect about 19 cases in children and 5 cases in adults (12 cases in Year 1 with a cohort of 1750 participants and 12 cases in Year 2 with a cohort of about 1662 participants). The overall incidence rate would be 7.1 per 1000 person–years with an exact Poisson 95% confidence intervals (CI) of (4.5; 10.6) and with a CI based on the normal approximation and accounting for the design effect of (3.3; 10.9).

#### Design effect

The enrollment was conducted by household. Each household could be considered as a cluster and this induced a design effect to account for the between-cluster variability when estimating CI of the incidence rates. The design effect measures the increase in the standard error of the incidence rate estimate due to the sampling design used and is given by D = 1 + (b–1)ρ, where ρ is the intracluster correlation (a measure of the rate of homogeneity within clusters) and b is the average number of individuals sampled per household. Here, b was assumed to be 3. Although in theory “ρ” can have a value up to 1, in practice values >0.4 are uncommon. A conservative estimate of >0.4 was used for this study. The design effect was then estimated at 1.8.

#### Analyses

Statistical analysis was performed on the per-protocol cohort that included all evaluable eligible participants. Demographic characteristics (age at Visit 1, sex, and number of participants enrolled per household) were summarized using descriptive statistics. The incidence proportion of cases confirmed virologically, cases confirmed only with RT-qPCR, and probable SDCs during the study period were calculated. As the correlation of the observations among individuals from the same household (i.e., clustering effect) was anticipated, these proportions were also estimated using a marginal logistic model using generalized estimating equations (GEE) accounting for correlated data. The 95% CI accounting for correlated data was computed for all estimated incidence proportions. GEE is the extension of the generalized linear models (i.e., standard statistical methodologies) for the analysis of correlated data such as clustered data or repeated measurements. However, given the small number of participants per cluster (household) in this study, the GEE method may downwardly bias standard error estimates. Therefore, if the estimated design effect was <1, then the classical logistic regression model not accounting for correlated data was used instead of GEE to estimate the incidence proportion and the 95% CI.

The clinical symptoms reported during SDCs were tabulated. The proportion (with 95% CI) of participants with a DENV antibody IgG positive result (ELISA) at Visit 1 was estimated by age group using the same methodology as for the incidence proportions. All statistical analyses were performed using Statistical Analysis Systems (SAS Institute) software (version 9.4).

## Results

Of the 500 individuals enrolled, 498 completed the study follow-up through Month 12 (Visit 2). One left the study area, and another withdrew consent. Almost one-third of the participants were adolescents and another one-third were adults aged up to 50 years (**Table 1**). Of the 500 individuals tested for anti-DENV IgG seropositivity at enrollment, 494 yielded available results and were included in the statistical analysis. Almost all study participants older than 9 years were anti-DENV IgG-positive (95.7%, 309/323) (**Table 2**).

**Table 1:** Sociodemographic characteristics of the participants

**Characteristics**	**N = 500**
Households, number	352
Sex, female, n (%)	272 (54.4)
Age (years)	
Mean (SD)	15.6 (12.6)
Median	12.0
Range	0.5–50
Age groups, n (%)	
6 months to <12 months	12 (2.4)
12 months–4 years	75 (15.0)
5–8 years	88 (17.6)
9–17 years	175 (35.0)
18–50 years	150 (30.0)
Number of participants enrolled per household, n′ (%)	
1	204 (58.0)
2	148 (42.0)

N, number of participants; n, number of participants in a given category; n′, number of households in a given category; SD, standard deviation.

**Table 2:** Proportion of DENV IgG+ participants by ELISA at Visit 1

	**n**	**Positive dengue (%)**	Estimated GEE (%)^*^	**95%CI†**
Overall	494	377 (76.3)	76.2	71.9–80.0
Age group				
6 months to <12 months^1^	12	1 (8.3)	8.3	1.2–41.3
12 months–4 years	74	23 (31.1)	31.2	21.7–42.7
5–8 years	85	44 (51.8)	51.5	40.8–62.0
9–17 years	173	160 (92.5)	92.0	86.4–95.4
18–50 years	150	149 (99.3)	99.3	95.4–99.99

n, number of participants with available results; CI, confidence interval; DENV, dengue virus; ELISA, enzyme-linked immunosorbent assay; GEE, generalized estimating equations; IgG, immunoglobulin G.

* Proportion estimated from GEE logistic regression model taking the clustering (the households) effect into account, except for¹ = (n/n) × 100 as the design effect is ≤1;

† based on the robust variance estimate from the GEE model except for¹ = Wald CI as the design effect is ≤1.

Unscheduled visits were reported between January and September 2018, except for February and June. No unscheduled visits were made in the last quarter of 2017 or 2018. Overall, 85% of parents readily answered monthly and weekly calls (the remaining 15% were home visits). The overall incidence proportion of virologically and only RT-qPCR confirmed dengue cases over the 16-month study period was 0.8 (95% CI 0.3–2.1).

**Figure 2:**
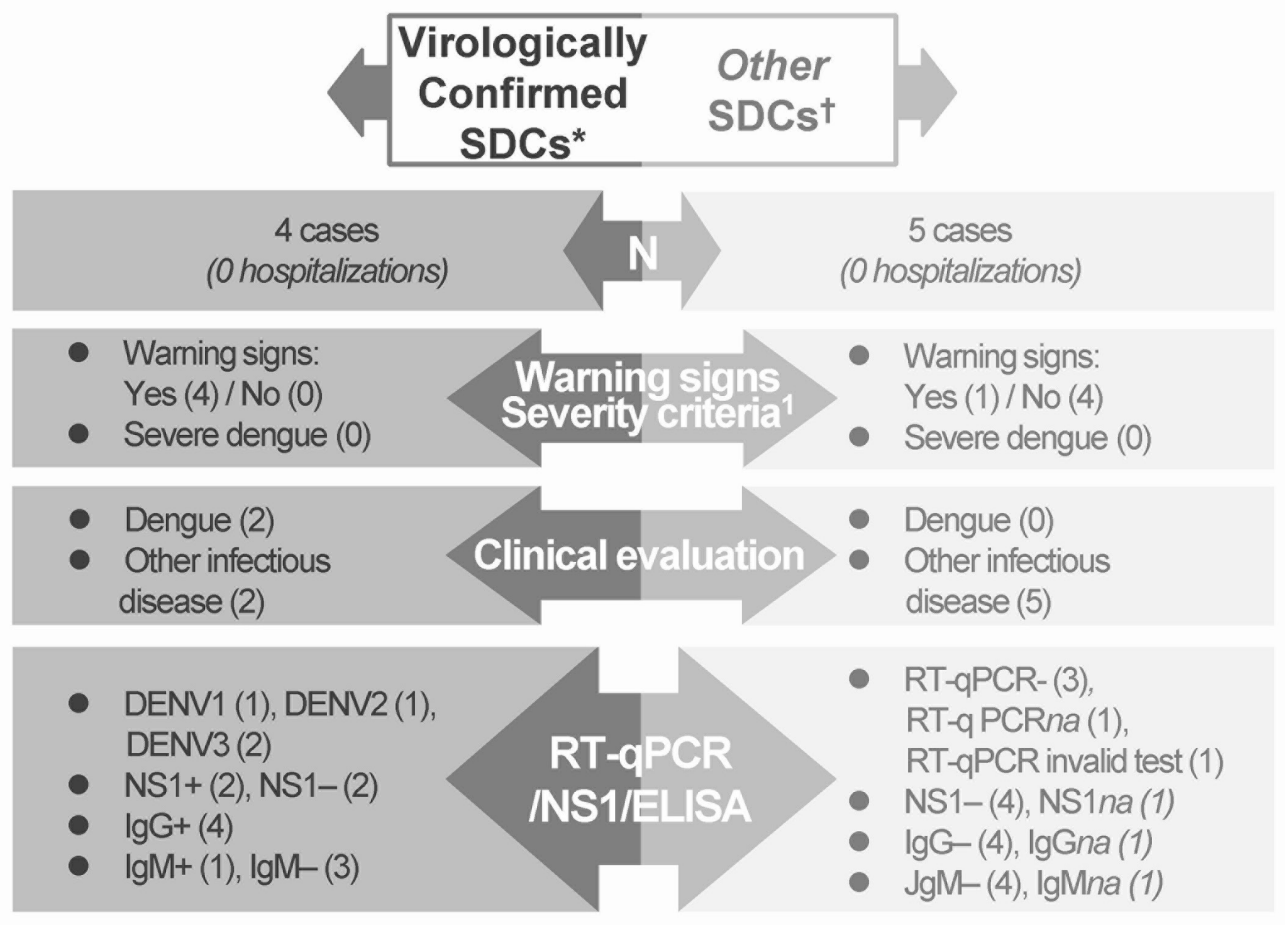
SDCs. *SDCs confirmed by RT-qPCR or NS1. To be classified with a SDC a study participant should have a body temperature of ≥38.0 °C within the past 8 days lasting from 36–48 h to 7 days, potentially accompanied by other signs of dengue that by the study investigator’s opinion could only be related to dengue; †SDCs not classified as confirmed virologically or probable. **Warning signs**. At least one of the following should be present: abdominal pain or tenderness, persistent vomiting, clinical fluid accumulation, mucosal bleeding, liver enlargement, increase in hematocrit concurrent with a rapid decrease in platelet count, lethargy, restlessness. **Criteria for severe dengue**. Dengue with at least one of the following: severe plasma leakage leading to shock, fluid accumulation with respiratory distress, severe bleeding, severe organ involvement, failure of heart and other organs. DENV, dengue virus; ELISA, enzyme-linked immunosorbent assay; ICT, immunochromatographic assay; IgG, immunoglobulin G; IgM, immunoglobulin M; *na,* not available; NS1, nonstructural protein 1; RT-qPCR, reverse-transcriptase quantitative polymerase chain reaction; SDC, suspected dengue case.

Nine SDCs were reported, 4 were reported from January to April 2018, and 5 from July to September 2018 and none required hospitalization. Four SDCs were confirmed virologically as being cases of dengue; the other 5 SDCs did not meet the criteria for virological confirmation. Two of the 4 dengue cases confirmed virologically were correctly diagnosed (**Figure 2**). These confirmed cases of dengue were in children aged 4, 5, 11, and 14 years. No probable dengue cases were recorded.

As shown in **Table 3**, all participants with SDCs developed fever and 2 participants among the 4 with SDCs confirmed virologically had a body temperature of ≥38.0 °C at the first (unscheduled) visit for dengue suspicion. The next most common symptoms were cough and headache (**Table 3**). No serious adverse events (SAEs) related to study procedures were reported.

**Table 3:** Clinical evaluation of patients with cases of dengue confirmed virologically and other SDCs

**Clinical symptoms**	Virologically confirmed dengue cases^*^ n = 4	**Other SDC† n = 5**	**Total n = 9**
At first visit for SDC			
Axillary body temperature, n (°C)			
<38.0	2	5	7
≥38.0	2	0	2
Symptoms recorded during the SDC episode, n			
Lasting fever^‡^	4	5	9
Cough	2	3	5
Headache	2	2	4
Nausea or vomiting	1	2	3
Abdominal pain	2	0	2
Nasal congestion	0	2	2
Retroorbital pain (eye pain)	1	0	1
Sore throat	1	0	1
Petechia	1	0	1
Other	3	2	5

n, number of cases; NS1, nonstructural protein 1; RT-qPCR, reverse-transcriptase quantitative polymerase chain reaction; SDC, suspected dengue case.

* Confirmed by RT-qPCR or NS1;

† SDC not virologically confirmed nor probable;

‡ body temperature ≥38.0 °C lasting from 36–48 h to 7 days in the past 8 days.

## Discussion

The present study was conducted to determine the incidence of dengue among household members aged 6 months to 50 years in a dengue-endemic municipality. Active surveillance was implemented to detect SDCs, in parallel with routine passive surveillance. All reported SDCs were detected by the active-surveillance process and all dengue cases confirmed virologically occurred from July to September, during the rainy summer monsoon season for the Philippines [22], a period known to be significantly associated with increased dengue incidence [23]. In countries with marked seasonality, the recruitment period should preferably occur outside the period of peak incidence of dengue, based on the local epidemiology of dengue in the past years. It should also preferably occur outside the holiday period if the investigator believes that families are more likely to leave the study area during this time. These periods should be described for each site and may be modified upon mutual agreement between the investigator and the central study team based on available epidemiological information.

Dengue was common in the study community, as demonstrated by the high IgG prevalence at enrollment, with nearly 4 of 5 study participants tested positive for DENV IgG. Consistent with previously published epidemiological data, almost all adults were seropositive for DENV IgG antibody at study enrollment, suggesting that most of the adult population in this area had been infected previously by dengue [5]. Moreover, DENV IgG seropositivity was detected in the vast majority of participants aged from 9 to 18 years, suggesting that most infections had occurred during childhood or early adolescence. The cases confirmed virologically were in children aged from 4 years to 14 years. These results are consistent with current epidemiological information that, in the Philippines, dengue is prominently observed in children and young teenagers (5–14 years of age) [5]. A previously reported active-surveillance cohort study, conducted in 2012, with 12 months of follow-up in about 1000 residents of Cebu City, the Philippines, recorded 13 SDCs confirmed by RT-PCR, 12 of which were in children aged <15 years [24]. A study that examined at dengue seroprevalence in 46 endemic sites in 13 Asian and Latin American countries found that the rate of infection varied greatly between countries, ranging from 1.7% in Singapore to 24.1% in the Philippines [25]. Seroconversion was observed in 50% of participants from the 44 sites by the age of 18 years [25]. The Mexican branch of the present study reported an overall seroprevalence of 19.4% (95% CI 14.5–25.6) suggesting that most of the population was naïve to dengue [26]. Another study, of a similar design, with 3300 participants among households in Brazil, found similarly high (76.2%) baseline seroprevalence rates, while only 23.3% of participants had reported a history of dengue [27]. These observations illustrate the heterogeneous nature of dengue transmission, with variation in the rate of infection across different regions of endemic countries.

Despite active (weekly) surveillance in our study, the incidence of virologically confirmed dengue was low. The detection of SDCs requires a sensitive definition. Our surveillance strategy was focused on the occurrence of an acute febrile illness lasting at least 36–48 h. This criterion to define a SDC requiring clinical and laboratory evaluation may have been too stringent in the context of the common use of antipyretics. In addition, atypical presentations of dengue have been described and deserve more attention [28,29,30].

Dengue infections may be asymptomatic in 75% overall [31]. These infections would not be detected by our study, which focused on symptomatic cases.

The Philippine dengue surveillance system depends mostly on cases in hospitalized patients with severe symptoms. For example, in 2010–2014, 93% of reported cases were in hospitalized patients [7]. Therefore, the sensitivity of the surveillance strategy in identifying acute infections is most likely suboptimal and should be considered inadequate for a country like the Philippines where dengue is highly endemic. Moreover, data show that dengue has become hyperendemic in most parts of the Philippines, with increasing incidence [24]. The highest epidemic incidence was recorded in 1998 with 60.9 cases per 100,000 population and 2.6% case fatality rate [5]. This study adds to the knowledge of the disease dynamics in the Philippines, which will help in the design of vaccine trials. To understand dengue transmission dynamics better, and to detect changes in clinical presentation, an intensive sentinel surveillance approach may be more appropriate as already used globally with influenza. Periodic screening of a representative group of individuals meeting the criteria of a sensitive case definition for dengue, sampled from a stable, endemic community, with an NS1 rapid test in conjunction with RT-qPCR, may improve our understanding of local transmission dynamics, and, in turn, contribute to both health systems policies and prevention approaches [32].

The findings presented here bring a modest contribution to our current knowledge of the dengue epidemiology in the Philippines, which, although a highly endemic area, has only limited dengue seroepidemiology data [5]. Existing evidence suggests that all 4 DENV serotypes circulate in the Philippines [5]. Additional studies in more Philippine communities should be conducted, possibly with longer follow-up. Future studies could further consider assessing the impact of socioeconomic and other individual or community-based characteristics. The case definition for dengue suspicion should still be based on fever, but with less stringent criteria regarding its duration than in the present study. This would add to the effort to close the Philippine dengue data gap in circulating DENV monitoring and the population’s serologic status [33].

The premature discontinuation of the study is the major limitation of the present data because the short follow-up did not allow us to (a) observe community trends, (b) characterize dengue incidence in an additional season, and (c) characterize the spatiotemporal distribution of the cases, among households and in the broader community. Moreover, the sample size calculation was not made at the individual country level, but on an overall international, multicenter study level. The present data might not be generalizable outside the study’s population.

GEE is an extension of the generalized linear model and is used for the analysis of correlated data, such as clustered data and is applicable because the participants in the present study were within households (i.e., clusters) and correlation of the observations between individuals from the same household (i.e., a clustering effect) was anticipated. The model as applied to the study has no covariates. Statistical methodologies developed for simple random samples were not selected as the primary method as they assume independence of the observations (i.e., the probability of infection for each participant is independent of any other). Ignoring the clustering of observations could result in underestimating the variance of the estimated proportion. A limitation of GEE is mainly due to the number of individuals per household (cluster). This is why we applied the rule that if the estimated design effect was <1, then the standard logistic model not accounting for correlated data was used.

Another limitation is that blood samples were collected at Visit 2, but could not be analyzed due to the termination of the study. Even though it was not one of the study’s objectives, the analysis of these samples would have assisted in identifying asymptomatic cases that may have occurred after study enrollment. Furthermore, baseline measurement of antibodies against all dengue serotypes would have helped differentiate monotypic from multitypic dengue cases. Atypical and asymptomatic cases and cases in those with fever lasting <2 days have been missed due to the fever criteria used to define a SDC.

## Conclusions

**Figure 3:**
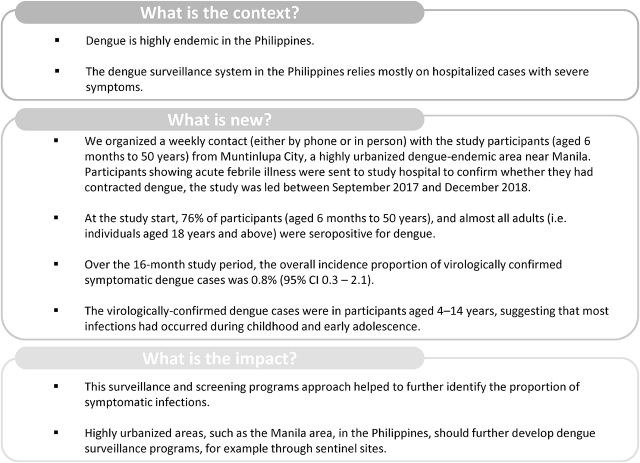
Plain language summary.

Highly urbanized dengue-endemic areas, such as metropolitan Manila, in the Philippines, must acquire the organizational potential to perform adequate routine surveillance, for example through sentinel sites. It is important to characterize the entire spectrum of clinical symptoms and identify community hot-spots of transmission. Those in these endemic areas should also have the opportunity to participate in research projects to further identify the proportion of asymptomatic viremic infections and to describe how these cases may contribute to transmission. A deeper knowledge of the dengue epidemic profile and transmission in highly epidemic areas would generate information of global epidemiological interest. Improvements in the surveillance of incident cases are warranted, coupled with improvements in clinical management strategies and efficient prevention strategies. The evidence presented here will enhance efforts in shaping effective surveillance strategies and in designing future dengue vaccine trials. **Figure 3** provides a plain language summary of the study findings.
